# *NKX2.1* run‐on mutation associated to familial brain–lung–thyroid syndrome

**DOI:** 10.1111/cge.13961

**Published:** 2021-03-29

**Authors:** Elena Cavaliere, Anna Jolanda Gortan, Nadia Passon, Dora Fabbro, Dario Marin, Miryam Carecchio, Federica Baldan, Sara Carmela Credendino, Rosa Gallo, Paola Cogo, Giuseppe Damante, Gabriella De Vita

**Affiliations:** ^1^ Academic Hospital of Udine Udine Italy; ^2^ Department of Neuroscience University of Padua Padua Italy; ^3^ Department of Medicine University of Udine Udine Italy; ^4^ Department of Molecular Medicine and Medical Biotechnology University of Naples Federico II Naples Italy

## Abstract

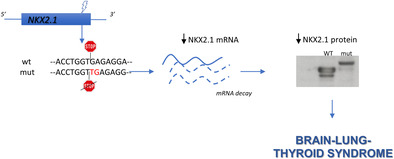

## PEER REVIEW

The peer review history for this article is available at https://publons.com/publon/10.1111/cge.13961.

To the Editor

*NKX2.1* gene encodes a homeobox transcription factor expressed in thyroid, lung, and several regions of ventral forebrain.^1^ Loss of function heterozygous mutations of *NKX2.1* cause the “brain–lung–thyroid” syndrome (BLTs), characterized by combinations of thyroid dysgenesis, infant respiratory distress syndrome and hyperkinetic movement disorders, mainly chorea.^2^ NKX2.1 haploinsufficiency is the likely pathogenetic mechanism of BLTs. Here, we describe a familial case of BLTs due to a *NKX2.1* run‐on mutation.

Patient III/1 (12 year old) was born at 34 weeks' gestational age. In the neonatal period, he required mechanical ventilation for pulmonary hypertension. During early infancy work‐up for frequent upper‐respiratory infections and short stature revealed subclinical hypothyroidism. Moreover, mildly delayed acquisition of motor milestones with poor coordination and sleep disturbances were reported. Our first assessment at 10 years of age revealed generalized chorea unstable gait and mild hypotonia. He underwent a normal brain MRI and psycho‐diagnostic assessment (quotient intelligence = 97; normal executive, attention and memory functions, while poor fine motor coordination and developmental coordination disorder, dysgraphia, and dysorthography). Therapy with tetrabenazine improved his movement disorder.

**FIGURE 1:**
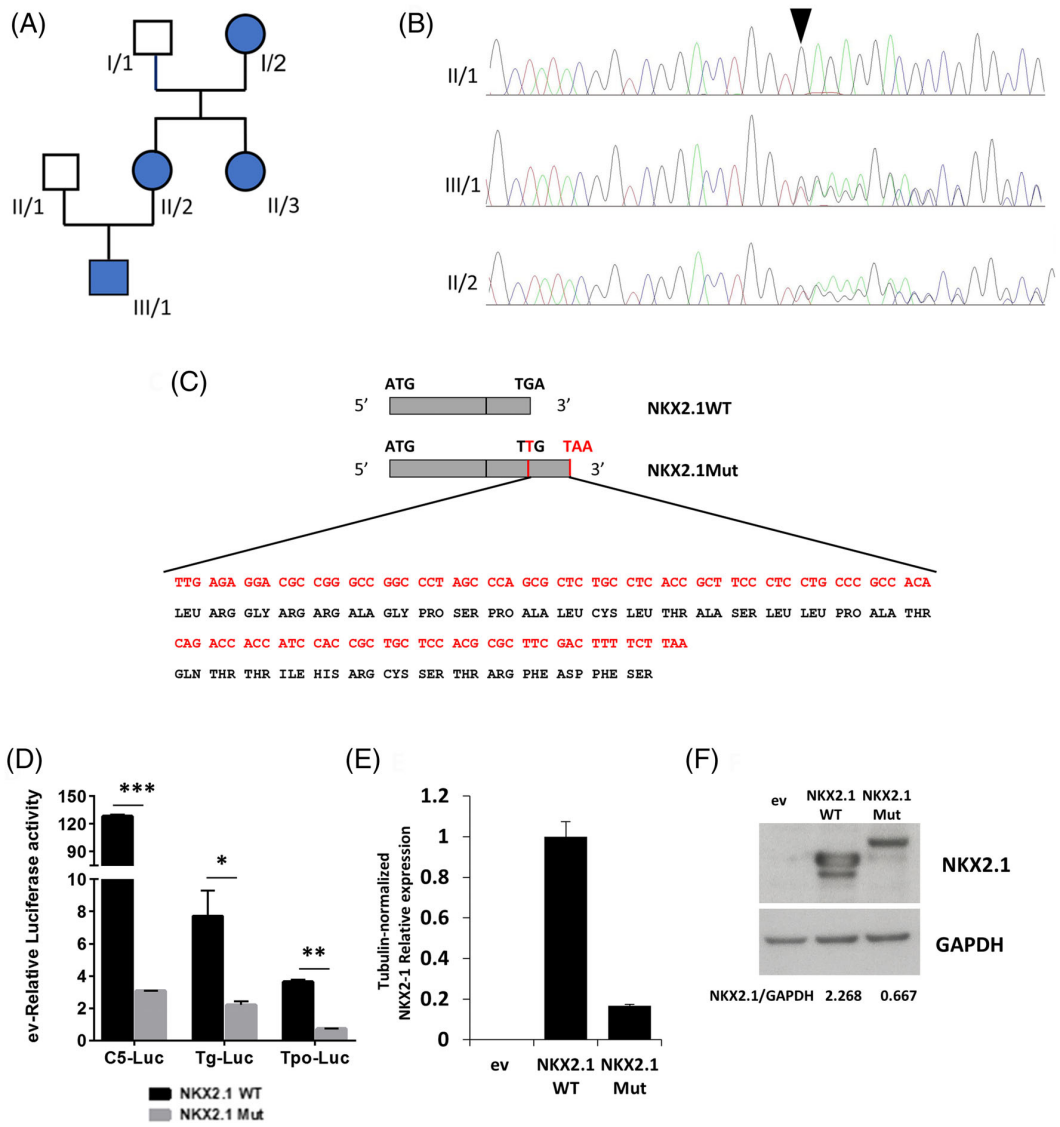
Pedigree and mutant *NKX2*.1 analysis. (A) Pedigree plot; the solid figure represents all affected family members (III/1, II/2; II/3 and I/2). (B) Sanger sequencing showing the *NKX2*.1 mutation (c.1204dupT) in the proband (III/1) and his mother (II/2), but not in the father (II/1). (C) Representation of the nucleotide duplication effects. (D) HeLa cells were transfected with WT or mutant (Mut) NKX2.1‐expressing vectors, or empty vector (ev) as control, alone or together with Luciferase (Luc) reporter vectors (C5‐Luc, Tg‐Luc and Tpo‐Luc). Luc activity is reported as folds of promoter activity in the presence of ev. (E) qRT‐PCR analysis of NKX2.1 WT and Mut mRNA levels. (F) Western blot showing NKX2.1 WT and Mut proteins, with GAPDH as loading control. Data are shown as means ± SD of three experiments. *p < 0.05, **p < 0.01; ***p < 0.001 [Colour figure can be viewed at wileyonlinelibrary.com]

The patient's mother (II/2, 43 year old) suffered of impaired gait and learning disability at childhood, and subclinical hypothyroidism at adulthood, while no respiratory distress was reported. Our examination revealed distal choreiform movements in the lower limbs. The patient's aunt (II/3, 51 year old) and grandmother (I/2, 71 year old) reported gait imbalance at childhood and were treated for hypothyroidism; however, no movement disorder nor respiratory distress were outlined. II/3 was also treated for mood disorders (see Figure 1A).

Patient III/1 was subjected to *NKX2.1* genetic testing. A heterozygous run‐on mutation (c.1204dupT; p.*402Leuext*37) was found in exon 3 (Figure 1B), duplicating the thymine in the first position of the stop codon that changed in a leucine‐coding codon. The resulting frameshift generated a tail of 36 additional amino acids at the protein C‐terminus (Figure 1C). Segregation analysis confirmed that all the affected relatives carried this mutation.

Mutant and wild type NKX2.1 cDNA were tested for their ability to transactivate NKX2.1‐responsive promoters. C5 is an artificial promoter containing 5X tandem‐repeated NKX2.1 binding sequence. Thyroglobulin (Tg) and thyroperoxidase (Tpo) are cellular NKX2.1‐regulated promoters. All promoters resulted significantly less activated by the mutant NKX2.1 respect to the WT (Figure 1D).

NKX2.1 mRNA was measured in transfected cells, showing that the mutant is expressed at lower levels compared to the WT (Figure 1E). Western blot of transfected cells, while confirming the increased molecular weight of mutant NKX2.1, shows that it is expressed at levels lower than the WT, with WT/mutant ratio similar to that of the corresponding mRNAs (Figure 1F). To our knowledge only another NKX2.1 run‐on mutation has been reported: a nucleotide change in the stop codon generating a 63 amino acid‐long C‐terminal tail.^3^ Interestingly, the patient had only neurological symptoms, suggesting a possible phenotypic heterogeneity in subjects harboring NKX2.1 run‐on mutations. According to the haploinsufficiency mechanism of BLTs pathogenesis, here we describe a new run‐on mutation causing downregulation of both NKX2.1 mRNA and protein. Thus, the major effect of this mutation occurs at the mRNA level, probably by its destabilization.

Nonstop‐mediated decay is a mechanism determining degradation of mRNAs lacking the in‐frame stop codon proposed to link run‐on mutations to phenotypic abnormalities in humans.^4^ Our data support this hypothesis.

## CONFLICT OF INTEREST

The authors declare no potential conflict of interest.

## Data Availability

Data availability statement: Data supporting the findings described in this study are available upon request. The NKX2.1 variant reported has been submitted to the ClinVar public database (VCV000984956.1).
